# ^Understanding dementia diagnosis: the ELSI‐Brazil Study^

**DOI:** 10.1002/alz70860_107542

**Published:** 2025-12-23

**Authors:** Andrew Christopher Miguel, Matheus Ghossain Barbosa, Cleusa P Ferri

**Affiliations:** ^1^ Universidade Federal de São Paulo (UNIFESP), São Paulo, São Paulo/SP, Brazil

## Abstract

**Background:**

Despite the increasing number of people with dementia (PWD), detection remains low worldwide. In Brazil, PWD is expected to triple by 2050 and diagnosis can be challenging. We aim to identify determinants that influence the likelihood of receiving or not receiving a diagnosis of Alzheimer's Disease dementia (ADD).

**Method:**

Data from 5,249 participants aged 60 years and older was analyzed, drawn from ELSI‐Brazil, a large nationally representative sample. Our methodology involved an algorithmic approach that integrated individual cognitive and functional status, as well as self‐reported information. Among those identified by the algorithm as having dementia, we conducted logistic regression analyses, using age, sex, marital status, educational attainment, ethnicity, urbanicity, comorbidities, utilization of healthcare services, and results from memory and verbal fluency tests as predictors of a self‐reported diagnosis of ADD.

**Result:**

We found no associations for sex, marital status, educational level, skin color, urbanicity, comorbidities, utilization of healthcare services and semantic fluency. However, our study indicated that both older age and better results from memory testing were significantly associated with diagnosis of ADD.

**Conclusion:**

Detection of dementia and the subsequent diagnosis were associated with older age. While prevalence increases in older age groups, this may also reflect a tendency for late diagnoses and a lack of screening among younger seniors. Regarding the association with memory testing, it is possible that the results were influenced by participants' memory bias and limitations inherent to the algorithm employed.

